# Expansion of acquired 16S rRNA methytransferases along with CTX-M-15, NDM and OXA-48 within three sequence types of *Escherichia coli* from northeast India

**DOI:** 10.1186/s12879-020-05264-4

**Published:** 2020-07-25

**Authors:** Jayalaxmi Wangkheimayum, Mohana Bhattacharjee, Bhaskar Jyoti Das, K. Melson Singha, Debadatta Dhar Chanda, Amitabha Bhattacharjee

**Affiliations:** 1 Department of Microbiology, Assam University Silchar, Silchar, India; 2 Department of Microbiology, Silchar Medical College and Hospital, Silchar, India

**Keywords:** 16S rRNA methyltranseferase, CTX-M-15, NDM, OXA-48, Aminoglycoside, *E.coli*

## Abstract

**Background:**

This study aimed to identify ten different 16S rRNA methyltransferase genes (*rmtA, rmtB, rmtC, rmtD, armA, rmtF, npmA, rmtH, rmtE* and *rmtG)* and their coexisting ESBL and carbapenemase with the emergence of three *E.coli* clones within a single study centre.

**Methods:**

A total of 329 non-duplicate *E.coli* isolates were studied to detect the presence of 16S rRNA methyltransferases along with β-lactamases (TEM, SHV, OXA, VEB, GES, PER,CTX-M types, NDM, OXA-48,VIM, IMP and KPC) using PCR assay. Horizontal transferability were validated by transformation and conjugation analysis. Plasmid incompatibility typing and MLST analysis was also performed.

**Results:**

A total of 117 isolates were found to be resistant to at least one of the aminoglycoside antibiotics. It was observed that 77 (65.8%) were positive for 16S rRNA methyltransferases. Among them thirty nine isolates were found to harbour only *bla*_CTX-M-15_, whereas combination of genes were observed in three isolates (*bla*_VEB_+ *bla*_CTX-M-15_ in 2 isolates and *bla*_PER_ + *bla*_CTX-M-15_ in 1 isolate). *bla*_NDM_ and *bla*_OXA-48_ like genes were found in 23 and 9 isolates, respectively. All the resistance genes were conjugatively transferable, and incompatibility typing showed multiple 16S rRNA methyltransferase genes were originated from a single Inc. I1 group. MLST analysis detected 3 clones of *E.coli*ST4410, ST1341 and ST3906.

**Conclusion:**

The present study identified emergence of three clones of *E.coli*, resistant to aminoglycoside -cephalosporin- carbapenem. This warrants immediate measures to trace their transmission dynamics in order to slow down their spread in clinical setting.

## Background

Aminoglycoside antibiotics have a wide spectrum of activity against both Gram positive and Gram negative bacteria and are used in combination with β-lactam antibiotics, especially third generation cephalosporins. However, emergence of multidrug resistance severely compromised this therapeutic option as organisms harbour multiple resistance determinants to make chemotherapeutic alternatives ineffective. 16S rRNA methylation is one of the major mechanisms of aminoglycoside resistance [1]. In India there are multiple reports of 16S rRNAmethyltrasferase genes (*armA,rmtA, rmtB, rmtC, rmtD* and *rmtF)* as published in various studies [2–4]. These enzymes are responsible for alteration of A site of 16S rRNA and reported across the globe [5–10]. These genes are often associated with horizontal transmission and expansion of aminoglycoside resistance in hospital settings along with other resistance determinants [11]. These 16S rRNA methyl transferases confer resistance to all clinically relevant aminoglycosides and in particular high level resistance to amikacin. In the present study we have investigated the presence of acquired 16 s rRNA rmehyltransferase genes along with other co-resistance determinants in the clinical isolate of *E. coli* in a tertiary referral hospital of northeastern part of India.

## Methods

### Bacterial strains

A total of 329 consecutive clinical isolates of *Escherichia coli* were obtained from Silchar Medical College and Hospital, India, during March 2018–February 2019 from the patients who were admitted or attended the clinic and associated with infection. The additional information of the isolates was presented in supplementary Table S1. Isolates were identified by VITEK® 2 compact system (Biomeriux, USA). The study was approved by Institutional ethical committee of both Silchar Medical College and Assam University,

### Antimicrobial susceptibility testing

Minimum inhibitory concentration (MICs) of isolates against aminoglycoside antibiotics namely; kanamycin, tobramycin, gentamicin, netilmicin, amikacin and streptomycin (Hi,Media,India) were determined by agar dilution method. Disc diffusion method was used for detection of susceptibility pattern towards imipenem (10 μg), meropenem (10 μg), cefepime (30 μg), aztreonam (30 μg) cefotaxime (30 μg), ceftazidime (30 μg), ceftriaxone (30 μg) and ciprofloxacin (5 μg). *E. coli* ATCC 25922 was used as control. Results were interpreted in accordance with Clinical and Laboratory Standards Institute (CLSI) guidelines 2017 [12].

### Molecular characterization of 16S rRNA methyltransferase genes

The isolates which were resistant to at least one of the aminoglycoside antibiotics tested were subjected to PCR assay targeting 16S rRNA methyl transferase genes namely; *rmtA, rmtB, rmtC, rmtD, rmtE, rmtF, rmtG rmtH armA* and *npmA* (Table 1). The PCR mixture composed of 12.5 μl Go Taq green Master mix (Promega, Madison, USA) 10 pmol of each primer and ~ 100 ng DNA template prepared by boiling centrifugation method (83 °C for 20mins). The PCR was conducted in Bio-RadT100™Thermal cycler with the conditions: Initial denaturation at 95 °C for 5 min, 32 cycles of denaturation at 95 °C for 30 s, annealing at 50 °C for 40 s, extension at 72 °C for 40 s and final extension at 72 °C for 7 min.

**Table 1:** Primers used in this study for amplification of 16S rRNA methyltransferase genes

Target primers	Sequence (Forward& reverse-5′-3′)	Amplicon size (bp)	Reference
*armA*	GGTGCGAAAACAGTCGTAGT	1153	[13]
	TCCTCAAATATCCTCTATGT		
*rmtA*	CTAGCGTCCATCCTTTCCTC	635	[13]
	TTTGCTTCCATGCCCTTGCC		
*rmtB*	GGAATTCCATATGAACATCAACGATGCC	756	[13]
	CCGCTCGAGTCCATTCTTTTTTATCAAGT		
*rmtC*	CGAAGAAGTAACAGCCAAAG	1000	[13]
	GCTAGAGTCAAGCCAGAAAA		
*rmtD*	TCATTTTCGTTTCAGCAC	744	[13]
	AAACATGAGCGAACTGAAGG		
*npmA*	CGGGATCCAAGCACTTTCATACTGACG	981	[13]
	CGGAATTCCAATTTTGTTCTTATTAGC		
*rmtE*	ATGAATATTGATGAAATGGTTGC	818	[14]
	TGATTGATTTCCTCCGTTTTTG		
*rmtF*	GCGATACAGAAAACCGAAGG	589	[15]
	ACCAGTCGGCATAGTGCTTT		
*rmtG*	AAATACCGCGATGTGTGTCC	250	[16]
	ACACGGCATCTGTTTCTTCC		
*rmtH*	AATGACCATTGAACAGGCAGC	760	[17]
	TCAAGCTGGGTTTGGCTGGA		

### Determination of co-existing ESBLs and carbapenemases

Further, all the 16S rRNA methyltransferase producing isolates were subjected to detection of ESBLs and carbapenemases. ESBL production was confirmed among by phenotypic screening containing 1 μg/ml of cefotaxime and ceftazidime followed by combined disc diffusion test as per CLSI guidelines (2017). PCR assay was carried out with primers specific for several commonly occurring beta – lactamases genes viz.; *bla*_TEM_, *bla*_SHV_, *bla*_OXA_, *bla*_VEB_, *bla*_GES_, *bla*_PER_ and *bla*_CTX-M_ types [18–20] *bla*_NDM_ [21], *bla*_OXA-48_, *bla*_VIM_, *bla*_IMP_ and *bla*_KPC_ [22]. Two separate multiplex PCR assays were performed. Reactions were run under the following conditions: initial denaturation at 95 °C for 5 min; 32 cycles of 95 °C for 1 min, 54 °C for 1 min and 72 °C for 1 min; and a final elongation at 72 °C for 7 min. The amplicons of the reaction with *bla*_CTX-M_ primers were further sequenced to identify the exact variant.

### Horizontal transferability assay

Total Plasmid content was extracted by QIAprep Spin Miniprep Kit (Qiagen, Germany) and isolated plasmids were subjected to transformation by heat shock method using *Escherichia coli* DH5α as recipient. Transformants were selected on to the Luria Bertani agar (Hi-Media, Mumbai,India) containing 2 μg/ml of kanamycin. Conjugation assay was performed where the isolates harbouring 16S rRNA methyltransferase genes, acted as donor and azide resistant *E.coli* J53 was used as recipient. Mating was performed where both the donor and recipient cells were cultured in LB broth (Hi-Media) till it attained optical density at 600 nm (OD_600_) of 0.8–0.9. Cells were mixed at a ratio of 1:5 donor-to-recipient and transconjugant was selected on LB medium (Hi Media,Mumbai,India) containing 2 μg/ml of kanamycin and 100 μg/ml of sodium azide. Transformants and transconjugants were further screened for the presence of 16S methyltransferase genes, ESBLs and carbapenemase genes as obtained in parent isolates.

### Plasmid incompatibility typing

All the transformants and transconjugants were selected for this experiment. Plasmids were characterized by PCR-based replicon typing (PBRT) to identify the different incompatibility (Inc.) types. PBRT targeted 18 different replicon types viz. FIA, FIB, FIC, HI1, HI2, I1-Iy, L/M, N, P, W, T, A/C, K, B/O, X, Y, F, and FIIA performing 5 multiplex PCR and 3 simplex PCR [23].

### Multilocus sequence typing (MLST)

Multilocus sequence typing (MLST) was carried out for all 16S rRNA methyl transferase producing *E.coli* using the protocols and conditions described on the *E. coli* MLST website (http://mlst.warwick.ac.uk/mlst/dbs/Ecoli/documents/primersColi-html). Sequence types were determined after analysing by using centre for genomic epidemiology MLST analysis tool (https://cge.cbs.dtu.dk/services/MLST/).

## Results

Antimicrobial susceptibility testing showed that majority of the isolates were susceptible to meropenem (296/329) followed by imipenem (293/329), cefepime (282/329) aztreonam (281/329), ceftazidime (279/329), cefotaxime (276/329) and ceftriaxone (276.329) whereas ciprofloxacin showed moderate activity (165/329). Among them, a total of 117 isolates were found to be resistant to at least one of the aminoglycoside antibiotics viz.; gentamicin, tobramycin, amikacin, netilmicin and kanamycin.

### Detection of 16S rRNA methyltransferase genes

Of 117 isolates, it was observed that 77 (65.8%) were positive for 16S rRNA methyltransferase genes. Among them, isolates harbouring *rmt C* (*n* = 12) were more in numbers followed by *armA* (*n* = 11), *rmtD* (*n* = 9), *rmt F* (*n* = 8), *rmtB* (*n* = 6), *npmA* (*n* = 5), *rmtH* (*n* = 4), *rmtE* (*n* = 1) and *rmtG* (*n* = 1). Combination of multiples genes were also observed in some isolates. These were as; *armA + rmtF* (*n* = 3), *rmtG*+ *rmtH* (*n* = 3), *armA* + *rmtF* (*n* = 3), *armA + rmtE* (*n* = 3), *rmtC*+ *rmtD* (*n* = 3), *armA* + *rmtA* (*n* = 2) and *armA + rmtF + rmtC + rmtD* (*n* = 3). The details of their distribution is given in Table 2.

**Table 2:** PCR assay results of 16S rRNA methyltransferase genes with co-existing ESBLs and Carbapenemase

Sl. no	16S rRNA methyltransferase genes harbouring isolates	Co-existing ESBLs		Co- existing carbapenemase		Sequence Types
1	*rmtC* (*n* = 12)	CTX-M-15(*n* = 9)	–	NDM (*n* = 1)	–	ST3906
2	*armA*(*n* = 11)	CTX-M-15(*n* = 3)	VEB (*n* = 1)	NDM (*n* = 2)	OXA-48 (*n* = 1)	ST4410
3	*rmtD*(*n* = 9)	CTX-M-15(*n* = 5)	–	NDM (*n* = 2)	OXA-48 (*n* = 1)	ST3906
4	*rmtF* (*n* = 8)	CTX-M-15(*n* = 2)	–	NDM (*n* = 1)	OXA-48 (*n* = 1)	ST1341
5	*rmtB* (*n* = 6)	CTX-M-15(*n* = 3)	–	NDM (*n* = 2)	OXA-48 (*n* = 2)	Unknown
6	*npmA* (*n* = 5)	CTX-M-15(*n* = 2)	VEB (*n* = 1)	NDM (*n* = 1)	–	Unknown
7	*rmtH* (*n* = 4)	CTX-M-15(*n* = 7)	PER (*n* = 1)	NDM (*n* = 3)	OXA-48 (*n* = 1)	ST4410
8	*rmtE* (*n* = 1)	CTX-M-15(*n* = 1)	–	NDM (*n* = 2)	–	Unknown
9	*rmtG*(*n* = 1)	CTX-M-15(*n* = 1)	–	NDM (*n* = 3)	OXA-48 (*n* = 1)	Unknown
10	*armA + rmtF*(*n* = 6)	CTX-M-15(*n* = 3)	–	NDM (*n* = 1)	–	ST4410
11	*rmtG+ rmtH* (*n* = 3)	CTX-M-15(*n* = 2)	–	NDM (*n* = 1)	–	ST4410
12	*armA + rmtE*(*n* = 3)	CTX-M-15(*n* = 1)	–	NDM (*n* = 2)	–	ST4410
13	*rmtC+ rmtD* (*n* = 3)	CTX-M-15(*n* = 1)	–	–	OXA-48 (*n* = 1)	ST3906
14	*armA + rmtA* (*n* = 2)	CTX-M-15(*n* = 1)	–	NDM (*n* = 1)	–	ST4410
15	*armA + rmtF + rmtC + rmtD*(*n* = 3)	CTX-M-15(*n* = 1)	–	NDM (*n* = 1)	OXA-48 (*n* = 1)	ST3906

### Co-existing ESBLs and Carbapenemases

Among the 16S RMTases producing isolates 42 of them were confirmed to be ESBL positive by phenotypic methods as thirty nine of them were harbouring only *bla*_CTX-M-15_ and a combination of *bla*_VEB_ + *bla*_CTX-M-15_ and *bla*_PER_ + *bla*_CTX-M_ was observed in two and in a single isolate respectively. Twenty three of them were *bla*_NDM_ positive and 9 isolates harboured OXA-48 like genes (Table 2).

### Horizontal transferability

All the resistance genes detected were conjugatively transferable and transformants carrying the 16SrRNAmethyl transferase genes along with ESBLs and carbapenemases could be selected in the media containing kanamycin. On performing PCR based replicon typing, it was observed that in all the isolates 16SrRNAmethyl transferase genes with coexisting ESBLs and carbapenemases were carried within I1 Inc. type plasmid.

### MLST analysis

While analysing multilocus sequence typing data it was found that *E.coli* isolates harbouring *rmtH,armA* and *rmtF* were of sequence type 4410. Additionally ST 1341 was found to carry *rmtF* gene. *E.coli* ST3906 was carrying *rmtC* and *rmtD*. Isolates harbouring *rmtB,rmtG,rmtE*and *npmA* gene were found to be of unknown Sequence types.

## Discussion

Aminoglycosides are potent and broad spectrum antibiotic which is often used to treat hospital acquired infections. Probably, this is the reason why different aminoglycoside modifying enzymes (AMEs) and methyl transferase enzymes are evolved in bacteria that are propagated and maintained among strains in hospital environment. These strains are needed to be detected and dissected in order to track their movement and potential lateral transfer of resistance determinants. The first 16 s rRNA methyl transferase gene *armA* was reported from Poland and subsequently *rmtA* gene was discovered from Japan [24]. Since then, it is reported from different part of the world. In India six variants are reported till date [2–4]. A recent study from Nepal has showed coexistence of DIM-1, NDM-1 and VIM-2 with *rmtB4* and *rmtF2* [25] where two novel variants of resistance genes were identified. In the current study we found ten different types of acquired 16S rRNA methyltransferase types along with CTX-M-15, VEB, PER, NDM and OXA-48 like beta-lactamases. Presence of co-existing ESBLs and carbapenemases are quite alarming as these would severely compromise combination therapy.

In our setting acquired 16S rRNA methyltransferase genes along with other beta-lactamase genes were found to be associated with Inc. I1 group of plasmid. Therefore, this broad host range plasmid was the genetic vehicle for horizontal transfer of aminoglycoside, cephalosporin and carbapenem resistance in this part of the India. In the current study three sequence types of *E. coli* were responsible for expansion and propagation of this multidrug resistant trait in this setting. In a similar study from US *E.coli* ST448 was found to harbour *rmtE1* type along with extended spectrum beta-lactamases [26]. However, the three sequence types of *E. coli* obtained in this study is not reported elsewhere to be associated with aminoglycoside resistance.

## Conclusions

The expansion of the three sequence types of *E.coli* ST4410, ST1341 and ST3906 appear to be potential risky local sequence types and of clinical concern that may complicate infection control program. Therefore, further investigations should be undertaken to trace the origin and evolution of these clones thereby preventing or atleast slowing down their spread.

## Supplementary information

**Additional file 1.**

## Data Availability

Available on request as per Government of India regulations.

## Abbreviations

MLST: Multilocus sequence typing
ESBL: Extended Spectrum Beta-lactamase
MIC: Minimum inhibitory concentration
