# Correction to: Expansion of acquired 16S rRNA methytransferases along with CTX-M-15, NDM and OXA-48 within three sequence types of *Escherichia coli* from northeast India

**DOI:** 10.1186/s12879-020-05326-7

**Published:** 2020-08-24

**Authors:** Jayalaxmi Wangkheimayum, Mohana Bhattacharjee, Bhaskar Jyoti Das, K. Melson Singha, Debadatta Dhar Chanda, Amitabha Bhattacharjee

**Affiliations:** 1 Department of Microbiology, Assam University Silchar, Silchar, India; 2 Department of Microbiology, Silchar Medical College and Hospital, Silchar, India

**Correction to: BMC Infect Dis 20, 544 (2020)**

**https://doi.org/10.1186/s12879-020-05264-4**

Following publication of the original article [1] we were notified of a few errors in Tables 1 and 2, highlighted below:

**Table 1:**
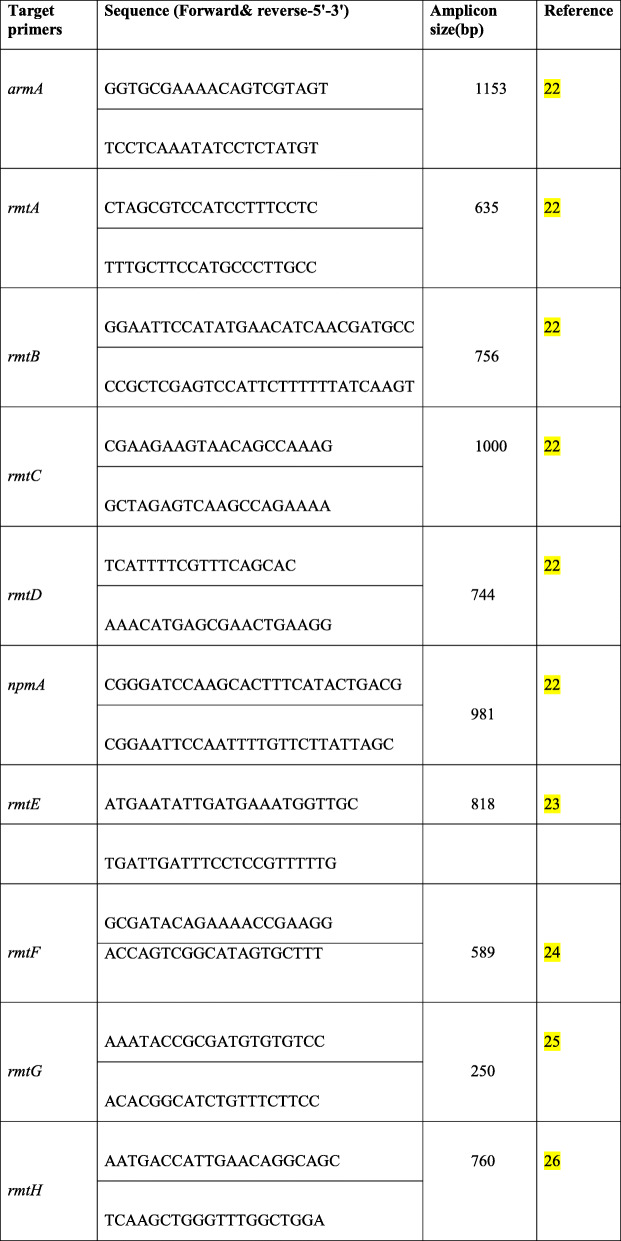
Primers used in this study for amplification of 16S rRNAmethyltransferase genes

**Table 2:**
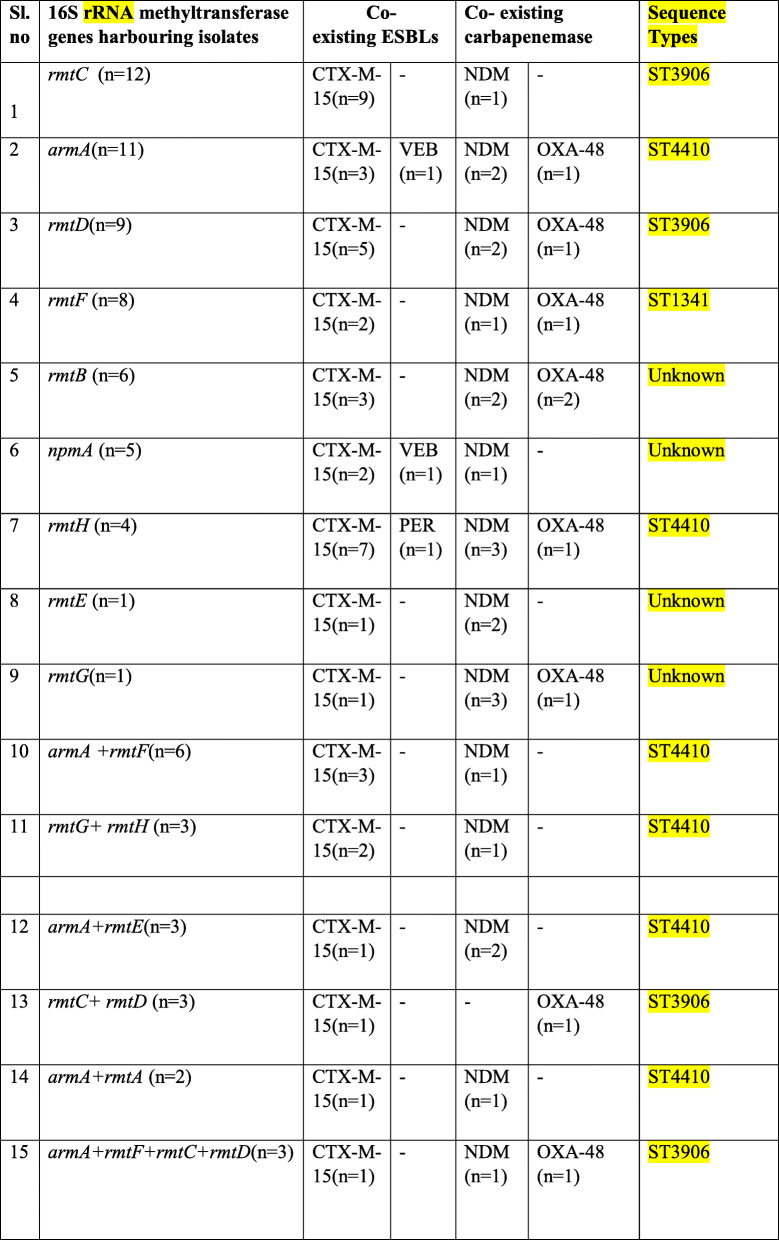
PCR assay results of 16S rRNA methyltransferase genes with co-existing ESBLs and Carbapenemase

The original article has been corrected.
