# Supplementary material for: Expansion of acquired 16S rRNA methytransferases along with CTX-M-15, NDM and OXA-48 within three sequence types of Escherichia coli from northeast India
Source: BMC Infect Dis. 2020 Jul 25;20:544. doi: 10.1186/s12879-020-05264-4 (PMC7382822; doi:10.1186/s12879-020-05264-4)
Supplement: Supplementary file 1 — Additional file 1. [file 12879_2020_5264_MOESM1_ESM.docx]

Supplementary table S1: Demographic information of the bacterial isolates

| **Sl.No** | **Sample ID** | **Clinical specimen** | **Patient age** | **Ward** |
| --- | --- | --- | --- | --- |
| 1 | **5489** | Pus | 25 | Medicine |
| 2 | **5487** | Pus | 49 | Surgery |
| 3 | **5491** | E Swab | 25 | ENT |
| 4 | **5475** | Urine | 3 | NICU |
| 5 | **5473** | Urine | 7 | Surgery |
| 6 | **5464** | Pus | 26 | Surgery |
| 7 | **5479** | Pus | 31 | Orthopaedic |
| 8 | **5488** | Sputum | 48 | Medicine |
| 9 | **5459** | Urine | 58 | Medicine |
| 10 | **5427** | Pus | 32 | Surgery |
| 11 | **5433** | Blood | 1 | Paediatric |
| 12 | **5484** | Urine | 4 | Paediatric |
| 13 | **5470** | Urine | 10 | Paediatric |
| 14 | **5463** | Urine | 32 | Medicine |
| 15 | **5454** | Urine | 4 | Paediatric |
| 16 | **5455** | Urine | 26 | Medicine |
| 17 | **5440** | Stool | 26 | Medicine |
| 18 | **5419** | Pus | 26 | Medicine |
| 19 | **5416** | Pus | 34 | Surgery |
| 20 | **5410** | Urine | 60 | Medicine |
| 21 | **5408** | Pus | 18 | Medicine |
| 22 | **5392** | Pus | 8 | Surgery |
| 23 | **5431** | Pus | 42 | Surgery |
| 24 | **5412** | Pus | 37 | ENT |
| 25 | **5475** | Urine | 3 | NICU |
| 26 | **5464** | Pus | 26 | Surgery |
| 27 | **5479** | Pus | 3 | Orthopaedic |
| 28 | **5488** | Sputum | 48 | Medicine |
| 29 | **5489** | Pus | 25 | Medicine |
| 30 | **5487** | Puss | 49 | Surgery |
| 31 | **5491** | E Swab | 25 | ENT |
| 32 | **5557** | Urine | 11 | Paediatric |
| 33 | **5592** | Urine | 21 | Surgery |
| 34 | **5602** | Pus | 16 | Orthopaedic |
| 35 | **5556** | Sputum | 3 | Paediatric |
| 36 | **5588** | Urine | 18 | ENT |
| 37 | **5555** | Pus | 70 | Orthopaedic |
| 38 | **5515** | Pus | 20 | Surgery |
| 39 | **5578** | Blood | 23 | Paediatric |
| 40 | **5591** | Aspirate fluid | 19 | Surgery |
| 41 | **5613** | Urine | 49 | Medicine |
| 42 | **5587** | Pus | 25 | Medicine |
| 43 | **5590** | Pus | 69 | Surgery |
| 44 | **5614** | Urine | 70 | Medicine |
| 45 | **5598** | Pus | 21 | Surgery |
| 46 | **5676** | Pus | 26 | Medicine |
| 47 | **5695** | Pus | 26 | Surgery |
| 48 | **5690** | Ear Swab | 26 | ENT |
| 49 | **5680** | Sputum | 34 | NICU |
| 50 | **5710** | Pus | 60 | Medicine |
| 51 | **5713** | Pus | 18 | Medicine |
| 52 | **5648** | Blood | 8 | Orthopaedic |
| 53 | **5711** | Pus | 42 | Medicine |
| 54 | **5659** | Blood | 37 | Medicine |
| 55 | **5661** | Blood | 3 | Surgery |
| 56 | **5677** | Sputum | 26 | Paediatric |
| 57 | **5681** | Pus | 3 | Paediatric |
| 58 | **5689** | Sputum | 48 | Paediatric |
| 59 | **5691** | Sputum | 25 | Paediatric |
| 60 | **5652** | Blood | 49 | Paediatric |
| 61 | **5672** | Pus | 25 | Surgery |
| 62 | **5678** | Pus | 11 | Medicine |
| 63 | **5675** | Urine | 21 | Medicine |
| 64 | **5670** | Urine | 16 | Surgery |
| 65 | **5683** | Pus | 25 | Medicine |
| 66 | **5703** | Pus | 49 | Medicine |
| 67 | **5705** | Pus | 25 | Surgery |
| 68 | **5701** | Sputum | 3 | Surgery |
| 69 | **5737** | Pus | 7 | ENT |
| 70 | **5731** | Sputum | 26 | NICU |
| 71 | **5709** | Pus | 31 | Surgery |
| 72 | **5707** | Pus | 48 | Orthopaedic |
| 73 | **5708** | Pus | 58 | Medicine |
| 74 | **6600** | Urine | 85 | Medicine |
| 75 | **6593** | Bile | 38 | Surgery |
| 76 | **6606** | Pus | 55 | Surgery |
| 77 | **6620** | Urine | 2 | Surgery |
| 78 | **6638** | Pus | 36 | Surgery |
| 79 | **6637** | Ascetic fluid | 57 | Medicine |
| 80 | **6609** | Blood | 6 M | NICU |
| 81 | **6656** | Ear Swab |  | Medicine |
| 82 | **6626** | Blood | 25 | Medicine |
| 83 | **6622** | Blood | 4 M | Paediatric |
| 84 | **6623** | Blood | 9 M | NICU |
| 85 | **6638** | Pus | 36 | Surgery |
| 86 | **6617** | Blood | 6 M | Surgery |
| 87 | **6636** | Pus | 55 | Surgery |
| 88 | **6633** | Pus | 55 | Surgery |
| 89 | **6644** | Urine | 70 | Medicine |
| 90 | **6628** | Blood | 6 M | Paediatric |
| 91 | **6640** | Throat Swab | 7 | ENT |
| 92 | **6653** | Pus | 17 | Surgery |
| 93 | **6659** | Pus | 9 | ENT |
| 94 | **6657** | Sputum | 13 | Medicine |
| 95 | **6666** | Urine | 66 | Medicine |
| 96 | **6670** | Pus | 20 | Medicine |
| 97 | **6682** | Pus | 34 | Surgery |
| 98 | **6699** | Urine | 66 | Paediatric |
| 99 | **6679** | Pus | 45 | Orthopaedic |
| 100 | **6710** | CSI | 3 M | Paediatric |
| 101 | **6716** | Urine | 7 | Paediatric |
| 102 | **6698** | Ear Swab | 65 | M ENT |
| 103 | **6678** | Pus | 49 | Surgery |
| 104 | **6661** | Blood | 6 M | Surgery |
| 105 | **7713** | Pus | 41 | Surgery |
| 106 | **7711** | Pus | 36 | Orthopaedic |
| 107 | **7699** | Pus | 44 | Surgery |
| 108 | **7709** | Pus | 35 | Surgery |
| 109 | **7662** | Sputum | 42 | Medicine |
| 110 | **7659** | Pus | 19 | Medicine |
| 111 | **7580** | Blood | 2 M | Surgery |
| 112 | **7608** | Blood | 27 M | NICU |
| 113 | **7633** | Blood | 19 M | NICU |
| 114 | **7663** | Pus | 5 | Surgery |
| 115 | **7651** | Urine | 66 | Orthopaedic |
| 116 | **7680** | Urine | 24 | Surgery |
| 117 | **7657** | Pus | 35 | Surgery |
| 118 | **7714** | Urine | 34 | Casualty |
| 119 | **7706** | Pus | 36 | Surgery |
| 120 | **7734** | Pus | 38 | Surgery |
| 121 | **7668** | Blood | 6 M | Surgery |
| 122 | **7687** | Urine | 6 | Medicine |
| 123 | **7690** | Urine | 48 | Medicine |
| 124 | **7712** | Urine | 11 | Surgery |
| 125 | **7648** | Blood | 38 | Orthopaedic |
| 126 | **7777** | Pus | 24 | Surgery |
| 127 | **7758** | Blood | 12 M | Paediatric |
| 128 | **7760** | Blood | 17 M | NICU |
| 129 | **7795** | Pus | 32 | Surgery |
| 130 | **7793** | Sputum | 27 | Surgery |
| 131 | **7761** | Urine | 70 | Surgery |
| 132 | **7755** | Pus | 30 | Surgery |
| 133 | **7739** | Urine | 7 | Medicine |
| 134 | **7741** | Sputum | 55 | NICU |
| 135 | **7745** | Pus | 70 | Surgery |
| 136 | **7753** | Pus | 60 | Surgery |
| 137 | **7726** | Blood | 24 M | Paediatric |
| 138 | **7733** | Blood | 3M | Paediatric |
| 139 | **7738** | Urine | 3Y 6 M | Paediatric |
| 140 | **7729** | Blood | 2 M | Paediatric |
| 141 | **7749** | Stool | 3 | Medicine |
| 142 | **7751** | PUS | 19 | Surgery |
| 143 | **7737** | PUS | 3 | Surgery |
| 144 | **8048** | Blood | 8 M | NICU |
| 145 | **8069** | Pus | 52 | Surgery |
| 146 | **8023** | Sputum | 44 | Paediatric |
| 147 | **8070** | Pus | 39 | Orthopaedic |
| 148 | **8044** | Pus | 40 | Surgery |
| 149 | **8042** | Pus | 67 | Surgery |
| 150 | **8085** | Urine | 1 | NICU |
| 151 | **8082** | Urine | 72 | Paediatric |
| 152 | **269** | Pus | 65 | Medicine |
| 153 | **265** | Pus | 30 | Surgery |
| 154 | **295** | Pus | 10 | Surgery |
| 155 | **285** | Pus | 24 | Medicine |
| 156 | **287** | Pus | 65 | Orthopaedic |
| 157 | **288** | Pus | 15 | Orthopaedic |
| 158 | **289** | Pus | 55 | Surgery |
| 159 | **300** | Pus | 60 | Surgery |
| 160 | **304** | Pus | 19 | Surgery |
| 161 | **306** | Endotracheal tube | 33 | Anastology |
| 162 | **274** | Blood | Day 4 | SNCU |
| 163 | **279** | Blood | Day 2 | SNCU |
| 164 | **311** | Pus | 24 | Surgery |
| 165 | **291** | Pus | 50 | Surgery |
| 166 | **270** | Urine | 47 | Surgery |
| 167 | **272** | Urine | 32 | Orthopaedic |
| 168 | **271** | Urine | 24 | Medicine |
| 169 | **268** | Pus | 60 | Surgery |
| 170 | **283 (i)** | Pus | 30 | Surgery |
| 171 | **283 (ii)** | Blood | 30 | Surgery |
| 172 | **371** | Urine | 40 | Surgery |
| 173 | **351** | Pus | 10 | Surgery |
| 174 | **357** | Pus | 25 | Surgery |
| 175 | **373** | Urine | 72 | Paediatric |
| 176 | **356** | Pus | 25 | Surgery |
| 177 | **387** | Pus | 25 | Surgery |
| 178 | **392** | Urine | 52 | Paediatric |
| 179 | **394** | Pus | 45 | ENT |
| 180 | **395** | Pus | 53 | Dermatology |
| 181 | **397** | Urine | 42 | Medicine |
| 182 | **398** | Pus | Day 12 | Medicine |
| 183 | **378** | Pus | 50 | Surgery |
| 184 | **379** | Pus | 38 | Orthopaedic |
| 185 | **383** | Urine | 24 | Surgery |
| 186 | **393** | Sputum | 75 | Medicine |
| 187 | **400** | Urine | 4 | Paediatric |
| 188 | **276** | Blood | Day 6 | Paediatric |
| 189 | **376** | Blood | Day 26 | Paediatric |
| 190 | **403** | Urine | 2 | Medicine |
| 191 | **382** | Urine | 10 | Paediatric |
| 192 | **972** | Urine | 52 | Surgery |
| 193 | **958** | Pus | 35 | Surgery |
| 194 | **962** | Sputum | 60 | Medicine |
| 195 | **956** | Urine | 27 | Casualty |
| 196 | **920** | Pus | 40 | Surgery |
| 197 | **955** | Blood | Day 2 | NICU |
| 198 | **990** | Pus | 10 | Surgery |
| 199 | **987** | CSF | 30 | Medicine |
| 200 | **986** | Sputum | 76 | Medicine |
| 201 | **985** | Urine | 72 | Surgery |
| 202 | **988** | Sputum | 60 | Medicine |
| 203 | **976** | Blood | 65 | Surgery |
| 204 | **966** | Pus | 55 | Surgery |
| 205 | **961** | Sputum | 50 | Medicine |
| 206 | **942** | Blood | Day 2 | Paediatric |
| 207 | **955** | Blood | Day 2 | SNCU |
| 208 | **1408** | Blood | 23 | Cardiology |
| 209 | **1562** | Blood | Day 3 | SNCU |
| 210 | **1575** | Urine | 22 | Orthopaedic |
| 211 | **1583** | Pus | 28 | Surgery |
| 212 | **1589** | Urine | 30 | Medicine |
| 213 | **1595** | Pus | 45 | Surgery |
| 214 | **1596** | Urine | 25 | Orthopaedic |
| 215 | **1597** | Pus | 52 | Surgery |
| 216 | **1600** | Urine | 11 | Paediatric |
| 217 | **1544** | Urine | 20 | Orthopaedic |
| 218 | **1546** | Urine | 14 | Orthopaedic |
| 219 | **1565** | Urine | Day 11 | Paediatric |
| 220 | **1563** | Urine | 52 | Medicine |
| 221 | **1555** | Urine | 48 | Medicine |
| 222 | **1533** | Blood | Day 3 | NICU |
| 223 | **1566** | Sputum | 54 | Medicine |
| 224 | **1569** | Pus | 50 | Dermatology |
| 225 | **1547** | Sputum | 50 | Medicine |
| 226 | **1549** | Pus | 50 | Surgery |
| 227 | **1556** | Sputum | 82 | ENT |
| 228 | **154** | Urine | 2 | NICU |
| 229 | **159** | Pus | 10 | Paediatric |
| 230 | **151** | Urine | 52 | Medicine |
| 231 | **158** | Sputum | 35 | Surgery |
| 232 | **152** | Pus | 60 | Surgery |
| 233 | **238** | Pus | 27 | Medicine |
| 234 | **236** | Blood | 40 | Orthopaedic |
| 235 | **235** | Blood | 5 | Orthopaedic |
| 236 | **232** | Urine | 2 | Surgery |
| 237 | **279** | Blood | 10 | Surgery |
| 238 | **285** | Stool | 52 | NICU |
| 239 | **212** | Pus | 35 | Paediatric |
| 240 | **215** | Pus | 60 | Medicine |
| 241 | **219** | Blood | 27 | Surgery |
| 242 | **230** | Blood | 40 | Surgery |
| 243 | **252** | Urine | 19 | Medicine |
| 244 | **254** | Blood | 14 | Orthopaedic |
| 245 | **259** | Stool | Day 2 | Orthopaedic |
| 246 | **1235** | Urine | Day 2 | Surgery |
| 247 | **1238** | Pus | 23 | Surgery |
| 248 | **1239** | Urine | Day 3 | NICU |
| 249 | **1230** | Pus | 22 | Paediatric |
| 250 | **1231** | Urine | 28 | Medicine |
| 251 | **1236** | Pus | 30 | Surgery |
| 252 | **1431** | Urine | 45 | Surgery |
| 253 | **1432** | Sputum | 25 | Medicine |
| 254 | **1435** | Pus | 4 | Orthopaedic |
| 255 | **1437** | Pus | 15 | Orthopaedic |
| 256 | **1440** | Blood | 27 | Surgery |
| 257 | **1441** | Blood | 25 | Surgery |
| 258 | **1442** | Urine | Day 2 | NICU |
| 259 | **1443** | Blood | Day 2 | Paediatric |
| 260 | **1445** | Stool | 23 | Medicine |
| 261 | **1446** | Urine | Day 3 | Surgery |
| 262 | **1447** | Pus | 22 | Surgery |
| 263 | **1448** | Urine | 28 | Medicine |
| 264 | **1449** | Urine | 30 | Orthopaedic |
| 265 | **1460** | Blood | 45 | Orthopaedic |
| 266 | **1461** | Stool | 25 | Surgery |
| 267 | **1465** | Urine | 19 | Surgery |
| 268 | **1469** | Pus | 12 | Paediatric |
| 269 | **1671** | Urine | 5 | Medicine |
| 270 | **1672** | Blood | 2 | Surgery |
| 271 | **1679** | Stool | 10 | Surgery |
| 272 | **1674** | Urine | 5 | Medicine |
| 273 | **1716** | Pus | 9 | NICU |
| 274 | **1712** |  | 11 | Paediatric |
| 275 | **1719** | Pus | 10 | Paediatric |
| 276 | **1751** | Urine | 17 | Medicine |
| 277 | **1761** | Pus | 2 | Surgery |
| 278 | **1781** | Pus | Day 2 | Surgery |
| 279 | **1798** | Urine | Day 2 | Medicine |
| 280 | **1751** | Blood | 23 | NICU |
| 281 | **1752** | Stool | Day 3 | Paediatric |
| 282 | **1891** | Urine | 22 | Paediatric |
| 283 | **1892** | Pus | 28 | Medicine |
| 284 | **1894** | Pus | 30 | Surgery |
| 285 | **1895** | Urine | 45 | NICU |
| 286 | **1860** | Pus | 25 | Paediatric |
| 287 | **1865** | Blood | 19 | Medicine |
| 288 | **1863** | Urine | 12 | Surgery |
| 289 | **1870** | Pus | 5 | Surgery |
| 290 | **1872** | Urine | 2 | Medicine |
| 291 | **1873** | Pus | 10 | Orthopaedic |
| 292 | **444** | Urine | 52 | Orthopaedic |
| 293 | **461** | Pus | 35 | Surgery |
| 294 | **469** | Urine | 60 | Surgery |
| 295 | **471** | Blood | 27 | Orthopaedic |
| 296 | **4418** | Urine | 40 | Orthopaedic |
| 297 | **4491** | Pus | 45 | Surgery |
| 298 | **4489** | Urine | 25 | Surgery |
| 299 | **4482** | Pus | 19 | Surgery |
| 300 | **4475** | Pus | 12 | Surgery |
| 301 | **4479** | Urine | 5 | Surgery |
| 302 | **4451** | Pus | 2 | Paediatric |
| 303 | **4456** | Blood | 10 | Medicine |
| 304 | **4432** | Urine | 52 | Surgery |
| 305 | **4419** | Pus | 35 | Surgery |
| 306 | **4421** | Urine | 60 | Medicine |
| 307 | **4428** | Pus | 27 | NICU |
| 308 | **4430** | Urine | 40 | Paediatric |
| 309 | **4493** | Pus | 16 | Medicine |
| 310 | **4496** | Urine | 21 | Surgery |
| 311 | **4499** | Urine | 13 | Surgery |
| 312 | **5555** | Pus | Day 2 | Medicine |
| 313 | **5418** | Urine | Day 2 | Orthopaedic |
| 314 | **519** | Pus | 23 | Orthopaedic |
| 315 | **5350** | Urine | Day 3 | Surgery |
| 316 | **5384** | Pus | 22 | Surgery |
| 317 | **5371** | Urine | 28 | Surgery |
| 318 | **5379** | Pus | 30 | Surgery |
| 319 | **5391** | Pus | 45 | Surgery |
| 320 | **5289** | Urine | 25 | Surgery |
| 321 | **5241** | Pus | 19 | Surgery |
| 322 | **2134** | Pus | 5 | Surgery |
| 323 | **5710** | Blood | 2 | Surgery |
| 324 | **5510** | Urine | 10 | Medicine |
| 325 | **5517** | Pus | 52 | NICU |
| 326 | **5518** | Urine | 35 | Paediatric |
| 327 | **5556** | Pus | 60 | Medicine |
| 328 | **5559** | Urine | 27 | Surgery |
| 329 | **5551** | Pus | 40 | Surgery |
